# Cytofluorographic and molecular identification of a CD8-positive, TCR-α/β-negative intraocular T cell lymphoma: a case report and review of the literature

**DOI:** 10.1186/1752-1947-1-114

**Published:** 2007-10-26

**Authors:** Alvaro D Saenz, Alexandra Amador, Brianna M Ruiz, Janet Davis, Phillip Ruiz

**Affiliations:** 1 Department of Pathology, University of Miami, Miami, Florida, USA; 2 Department of Ophthalmology, University of Miami, Miami, Florida, USA; 3 Department of Surgery, University of Miami, Miami, Florida, USA

## Abstract

**Introduction:**

Cytofluorographic and molecular techniques are effective adjuncts in diagnosing intraocular lymphoma. Primary intraocular lymphoma is an uncommon entity predominantly of B cell origin and rarely with a T cell phenotype. The aim of the present paper is to report a case of a CD8-positive, TCR-α/β-negative intraocular T cell lymphoma and review the literature.

**Case presentation:**

T cell neoplasia was detected based on flow cytometric demonstration of an abnormal T cell population and polymerase chain reactions for immunoglobulin and T-cell receptor rearrangements demonstrating evidence of monoclonality. Flow cytometry revealed a T cell population aberrantly expressing T-cell lineage markers. This T cell population expressed CD2, bright CD3, CD8, bright CD7, CD38, CD69, and variable CD25. T-cell receptor γ gene rearrangement studies demonstrated evidence of T-cell gene rearrangement confirming that the T cells were monoclonal.

**Conclusion:**

We herein report the rare case of a TCR α/β-negative CD8+ intraocular T-cell lymphoma suggestive of gamma/delta origin diagnosed by flow cytometry and polymerase chain reaction.

## Introduction

The occurrence of intraocular lymphoma can be divided into metastatic lymphoma to the eye usually presenting as uveal small cell non Hodgkin lymphoma, or primary intraocular lymphomas (PIOL) that characteristically are vitreoretinal large B cell variety [1]. The majority of PIOL's, a subset of primary central nervous system lymphoma (PCNL), develop central nervous system involvement whereas only a minority of PCNL's have intraocular disease [2,3]. PIOL has a broad age range, is more frequent in women, typically is bilateral, and has a propensity to mimic benign inflammatory diseases. Immunodeficiency and immune dysregulation are predisposing risk factors [1,3]. We have previously shown that the cytomorphologic diagnosis of PIOL can be markedly enhanced by flow cytometric analysis of cell populations in vitreous fluid [4].

The vast majority of intraocular lymphomas are composed of B cells with T cell lymphomas being particularly rare. Patients with the latter disease sometimes have a history of mycosis fungoides, systemic T cell lymphoma/leukemia, acquired immunodeficiency syndrome, or infection with human T cell lymphotropic virus type I [1]. In a series study by Coupland SE et al, some T cell lymphomas in the periorbital or orbital region were CD8+ [5]. In general, most systemic T cell lymphomas express the α/β T cell receptor (TCR) whereas a minority has the γ/δ TCR [6]. However TCR rearrangement in ocular T cell lymphomas has not been well studied or described.

Herein, we report a case of an intraocular CD8+, TCR-α/β-negative T-cell lymphoma without apparent systemic, cutaneous, or central nervous system involvement, diagnosed by flow cytometry and molecular assessment of antigen receptor rearrangement.

## Case presentation

An 83-year old Hispanic woman presented to Bascom Palmer Eye Institute with loss of vision in the left eye, floaters and flashing lights for one to three months. Her past medical history was significant for hypertension, cardiomyopathy, chronic bronchitis, and dementia with psychosis controlled with haloperidol and evaluated with an MRI examination of the brain one month previously that had revealed no mass lesions. A scaly rash had been present on the left side of the face, arm, and leg for over 1 year for which she had consulted three dermatologists without a definite diagnosis; it had cleared substantially when she took an antibiotic for a gastrointestinal infection, but was still present. The lesion was not biopsied.

Ocular examination revealed 20/30 vision in the right eye and 20/400 in the left eye. The vitreous fluid in the left eye contained cells and there was consideration of an old, decolorized vitreous hemorrhage or an inflammatory reaction. There was no anterior segment inflammation. A small white fibrotic macular lesion was barely visible.

Vision improved to 20/80, but the vitreous cells increased in the periphery these findings suggested the presence of a lymphoproliferative disorder and the diagnosis of intraocular lymphoma was considered 5 months after the initial examination. Pars plana vitrectomy was performed and the vitreous was sent for flow cytometry and cytology studies. The macular lesion was unchanged; there were no other retinal lesions. Vision improved slightly to 20/70 two weeks after surgery.

Evaluation with her primary care physician revealed a white blood cell count of 4.8 × 100 cells/mm^3, absolute lymphocyte count 400 cells/mm^3, hemoglobin 12.0 gm/dl, hematocrit 35.7%, and platelets 127,000/mm^3. The patient refused additional evaluation and was alive 7 months after the surgery.

## Materials and methods

Vitreous humor was collected using a pars plana vitrectomy methodology with cutting and aspiration into a syringe. The specimens were immediately submitted to the pathology department for processing. Specimens were placed in Roswell Park Memorial Institute Media (RPMI, Mediatech, Herendon, VA) for flow cytometry and 95% ethanol for cytologic analysis.

0.5 cc of the cytology specimen of undiluted vitreous was pipetted into a cytospin chamber and spun at 1000 rpm for five minutes with the concentrated cells then fixed onto a glass slide. The specimen was subsequently stained with a Papanicolau procedure [7].

The diluted flow cytometry specimen was subjected to red cell lysis with ammonium chloride. Cell counts were obtained with a hemocytometer, and cell viability was assessed using propidium iodide methodology. A limited number of cells were available (1.5 × 10^5^) and aliquots were stained using a four color technique that utilizes monoclonal antibodies directly conjugated to either fluorescein, phycoerythrin, peridinin chlorophyll protein, or allophycocyanin. The scope of hematopoietic antigens examined for leukemia-lymphoma analysis in various four color combinations included CD45, TCR α/β, CD2, CD3, CD4, CD5, CD7, CD8, CD56 CD19, CD20, CD22, CD23, CD79a, kappa, lambda, CD11c, CD13, CD14, CD64, TdT (Supertechs, Inc, Bethesda, Md), CD10, CD38, CD25, CD69, HLADR, and CD138. Unless specified, all antibodies were purchased from Becton Dickinson (San Jose, CA). The cells were analyzed using a Becton-Dickinson FACSCalibur™ flow cytometer (Becton Dickinson, San Jose, CA).

Multiplex PCR reactions for Antigen Receptor (Immunoglobulin and T-cell receptor) rearrangements were performed as previously described [8]. Reagents from Invivoscribe Technologies, Inc. were used as per manufacturer's directions. DNA was purified from the CSF using Qiamp spin columns (Qiagen, Inc. Valencia, CA), quantitated and amplified using a ABI Geneamp 9700 thermal cycler (Applied Biosystems, Foster City, CA), following the thermal cycler profile unique to each assay. Detection was performed using an ABI 3100-Avant Genetic Analyzer. Fragment analysis using GeneMapper v 3.7 software was used to detect the fluorescently labeled amplified products. DNA from normal polyclonal populations gave a unique Gaussian distribution of amplified products in the expected size range for the primers used. Monoclonal populations gave unique, single sized peaks, with or without a significant polyclonal background.

## Results

**Figure 1:**
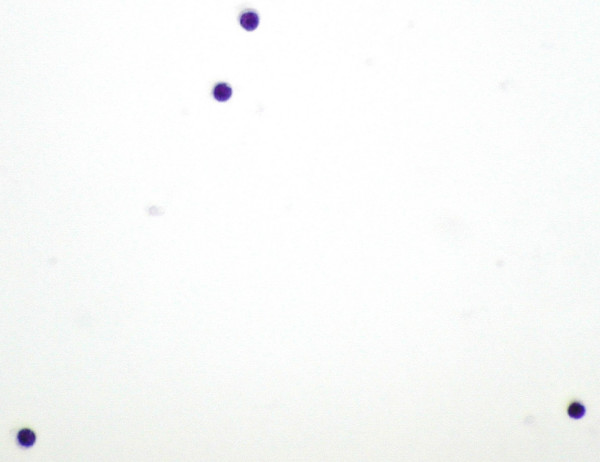
Papanicolaou stain, 600×. The corresponding flow cytometry cytospin has low cellularity and consists of small medium sized non-atypical mononuclear cells with minimal cytoplasm and a bland round nucleus consistent with lymphocytes.

**Figure 2:**
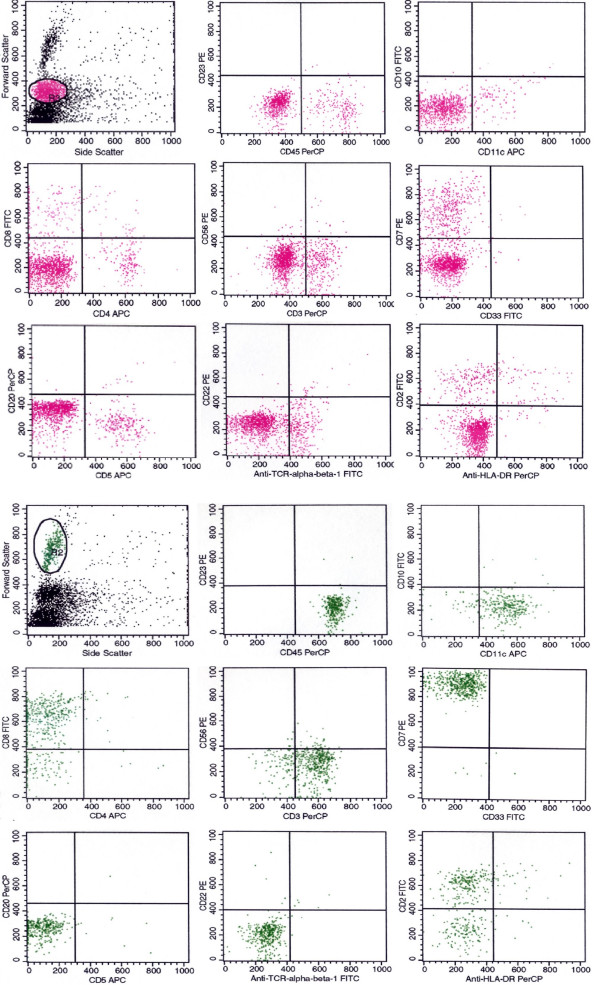
Flow cytometry histograms showing gating of small (red) and larger (green) cell populations. The larger cell population is primarily composed of atypical T cells.

Cytologic examination demonstrated few unremarkable mononuclear cells that varied in size (small, midsized) (Figure 1) and which mostly represented unremarkable lymphocytes. Forward and side scatter analysis revealed two distinctly sized agranular cell populations, each which was independently gated and analyzed. Flow cytometry multiparameter analysis of the gated cell populations revealed a distinct prominent T cell population that aberrantly expressed T-cell lineage markers. The larger sized cell population predominantly showed a T cell phenotype and expressed CD2, bright CD3, CD8, bright CD7, CD38, CD69, and variable CD25 (Figure 2). These cells failed to express CD4, CD5, or TCR-α/β. Normal T cells within the smaller sized cell gate expressed normal T cell lineage markers at typical intensities; B cells, and myelomonocytic cells were also identified. Overall, this phenotype was interpreted as an atypical T cell population suggestive of a T cell lymphoproliferative process.

**Figure 3:**
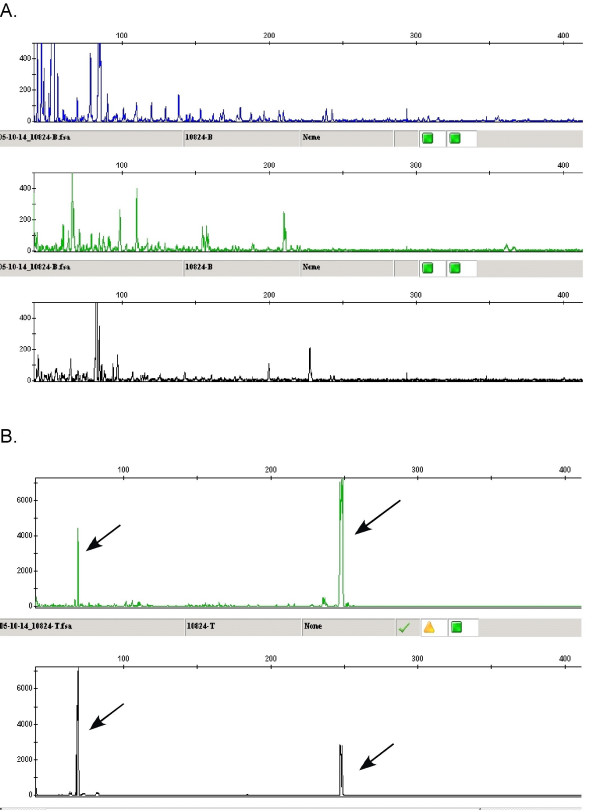
Multiplex molecular analysis of antigen receptors for B cell (A) and T cell (B) showing monoclonal spikes with the T cell primers (see arrows).

The immunoglobulin κ light-chain and heavy chain did not show any B cell monoclonality. T-cell receptor γ gene rearrangement studies demonstrated evidence of T-cell gene rearrangement (Figure 3) compatible with the T cells being monoclonal.

## Discussion

Intraocular T cell neoplasms are relatively uncommon and account for less than 1% of intraocular lymphomas. Patients typically have a history of either mycosis fungoides, systemic T cell lymphoma/leukemia, acquired immunodeficiency syndrome, or infection with human T cell lymphotropic virus type I [1]. In one study of 7 cases of orbital and periorbital T cell lymphoproliferative processes [5], 3 cases were CD8+, however, two of these cases were systemic tumors involving the eye and the remaining case was a polyclonal intraocular T cell population with a different phenotype than the one we have presented. In general, most systemic T cell lymphomas display surface α/β T cell receptor (TCR) whereas a minority express the γ/δ TCR [10]. However TCR rearrangement molecular analysis in ocular T cell lymphomas has not been well studied.

In the present case, vitreous fluid cytology, a traditional study for the diagnosis of intraocular lymphoma, did not reveal markedly atypical cells. However, multiparameter flow cytometry analysis demonstrated a distinct prominent T-cell population aberrantly expressing T-cell lineage markers. This T-cell population expressed CD2, bright CD3, CD8, CD7, bright CD38, CD69, and variable CD25. This T-cell population was negative for CD4, CD5, CD56, and TCR-α/β. PCR analysis of the flow cytometry cell mixture, by showing T-gamma gene rearrangement demonstrated the monoclonal nature of the T cells. By comparison, the admixed B cells did not show light chain gene rearrangement.

Although surface expression of TCR-γ/δ could not be confirmed directly because of insufficient specimen, its presence was strongly inferred from the lack of TCR-α/β expression. The patient lacked risk factors for systemic T cell neoplasia including ethnicity, geographical habitat, or immunodeficiency (e.g., HIV and HTLV). A brain MRI excluded a primary central nervous system primary, the peripheral blood cell count and smear did not reveal any atypical cells, and clinical examination did not reveal any skin lesions suspicious for mycosis fungoides, lymphadenopathy, or organomegaly. The scaly skin lesion improved with antibiotics. Thus, at this level there was no evidence of systemic disease. The patient was made aware of the findings but refused further work up and a bone marrow biopsy was not performed. Accordingly, we can only speculate on the primary or metastatic nature of the disease but the clinical and laboratory findings were indicative of a primary T-cell lymphoma suggestive of gamma/delta origin.

Gamma delta T cells display a restricted distribution mainly present in the spleen and certain epithelial sites. They are not MHC restricted, can secrete a variety of cytokines and can possess cytotoxic activity. They are essential in immunity against certain organisms and can be involved in autoimmune disorders, diabetes, asthma, and malignancy. Prototypical gamma/delta T-cell neoplasms include hepatosplenic gamma/delta T-cell lymphoma, mucocutaneous gamma/delta T cell lymphoma, and gamma/delta T-cell large granular lymphocyte (LGL) leukemia. Hepatosplenic gamma/delta T-cell lymphoma occurs in young males and has a dismal prognosis. It presents with hepatosplenomegaly, cytopenias, bone marrow and peripheral blood involvement, and no lymphadenopathy. The malignant cells demonstrate sinusoidal infiltration of the spleen, liver, and bone marrow. Their immunophenotype is usually CD2+, CD3+, CD4-, CD5-, CD7+, CD8 variable, CD16+, and CD56+ [6,8-10]. Mucocutaneous gamma/delta T cell lymphoma distinctly presents with skin or mucosal lesions without splenic or hepatic involvement and variable peripheral blood infiltration. Survival is worse than their TCR-α/β mucocutaneous counterparts and varies from aggressive to an indolent course. The typical immunophenotype is similar to gamma/delta hepatosplenic lymphoma with CD3+, CD7-, and variable NK related antigen expression [5,10]. Gamma/delta large granular lymphocyte (LGL) leukemia is usually an indolent disease presenting with clonal peripheral blood gamma/delta T cell lymphocytosis, neutropenia, recurrent bacterial infection, and bone marrow involvement. The malignant cells are usually CD2+, CD3+, and CD4-. Expression of CD8 and, CD5 are more frequent than in the other gamma/delta T cell lymphomas. This disease has a much better prognosis than the other two gamma/delta tumors [6,9].

## Conclusion

As previously mentioned, we have indirect evidence that the present case was in fact a true, somewhat indolent gamma/delta T-cell neoplasm, with no clinical evidence supporting systemic disease. The phenotype of the tumor would not be completely compatible with the less aggressive gamma/delta LGL lymphomas since the cells lacked CD5 expression nor was there neutropenia or recurrent bacterial infections. The patient's neoplasm was CD7+ and no malignant skin lesions were identified thus the probability of it representing a metastatic cutaneous gamma/delta T-cell lymphoma is unlikely. The patient's tumor immunophenotype is similar to hepatosplenic gamma/delta T-cell lymphoma, although it was negative for CD56 which is usually present in these neoplasms; moreover, the patient does not fit the profile of a young ill male with organomegaly. Taken together, our evidence supports this could represent a true primary intraocular gamma/delta CD8+ T-cell lymphoma although exact gamma/delta tumor categorization is not feasible.

## Consent

Written informed patient consent was obtained for publication.

## Competing interests

The author(s) declare that they have no competing interests.

## Authors' contributions

AS drafted the manuscript and participated in the design of the study. AA, BR carried out the molecular analysis and participated in the design of the study. JD participated in the design of the study. PR conceived of the study, and participated in its design and coordination and helped to draft the manuscript. All authors read and approved the final manuscript.
